# Roles of protein post-translational modifications in glucose and lipid metabolism: mechanisms and perspectives

**DOI:** 10.1186/s10020-023-00684-9

**Published:** 2023-07-06

**Authors:** Yu-Hang Yang, Ri Wen, Ni Yang, Tie-Ning Zhang, Chun-Feng Liu

**Affiliations:** Department of Pediatrics, Shengjing Hospital of China Medical University, No.36, SanHao Street, Liaoning Province, Shenyang City, 110004 China

**Keywords:** Post-translational modification, Glucose metabolism, Lipid metabolism, Metabolic disease

## Abstract

The metabolism of glucose and lipids is essential for energy production in the body, and dysregulation of the metabolic pathways of these molecules is implicated in various acute and chronic diseases, such as type 2 diabetes, Alzheimer’s disease, atherosclerosis (AS), obesity, tumor, and sepsis. Post-translational modifications (PTMs) of proteins, which involve the addition or removal of covalent functional groups, play a crucial role in regulating protein structure, localization function, and activity. Common PTMs include phosphorylation, acetylation, ubiquitination, methylation, and glycosylation. Emerging evidence indicates that PTMs are significant in modulating glucose and lipid metabolism by modifying key enzymes or proteins. In this review, we summarize the current understanding of the role and regulatory mechanisms of PTMs in glucose and lipid metabolism, with a focus on their involvement in disease progression associated with aberrant metabolism. Furthermore, we discuss the future prospects of PTMs, highlighting their potential for gaining deeper insights into glucose and lipid metabolism and related diseases.

## Introduction

Glucose and lipid metabolism, the main source of energy, is critical for the physiological functions of all tissues and organs (Chen et al. 2019). Dysregulation of glucose and lipid metabolism is a risk factor for many acute and chronic diseases, such as type 2 diabetes, Alzheimer’s disease (AD), atherosclerosis (AS), obesity, tumor, and sepsis (Cheng et al. 2016; Garcia et al. 2023; Gasbarrino et al. 2023; Takeuchi et al. 2023; Yassine et al. 2022). Glucose and lipid metabolism in the body is regulated by various proteins, including key enzymes. Any factor that affects these proteins may influence the metabolic processes. Recently, studies have confirmed that post-translational modifications (PTMs) participate in the metabolic processes of glucose and lipids and have a critical impact on diseases arising from aberrant glucose and lipid metabolism (Sawant et al. 2022; Stocks and Zierath 2022).

**Fig. 1:**
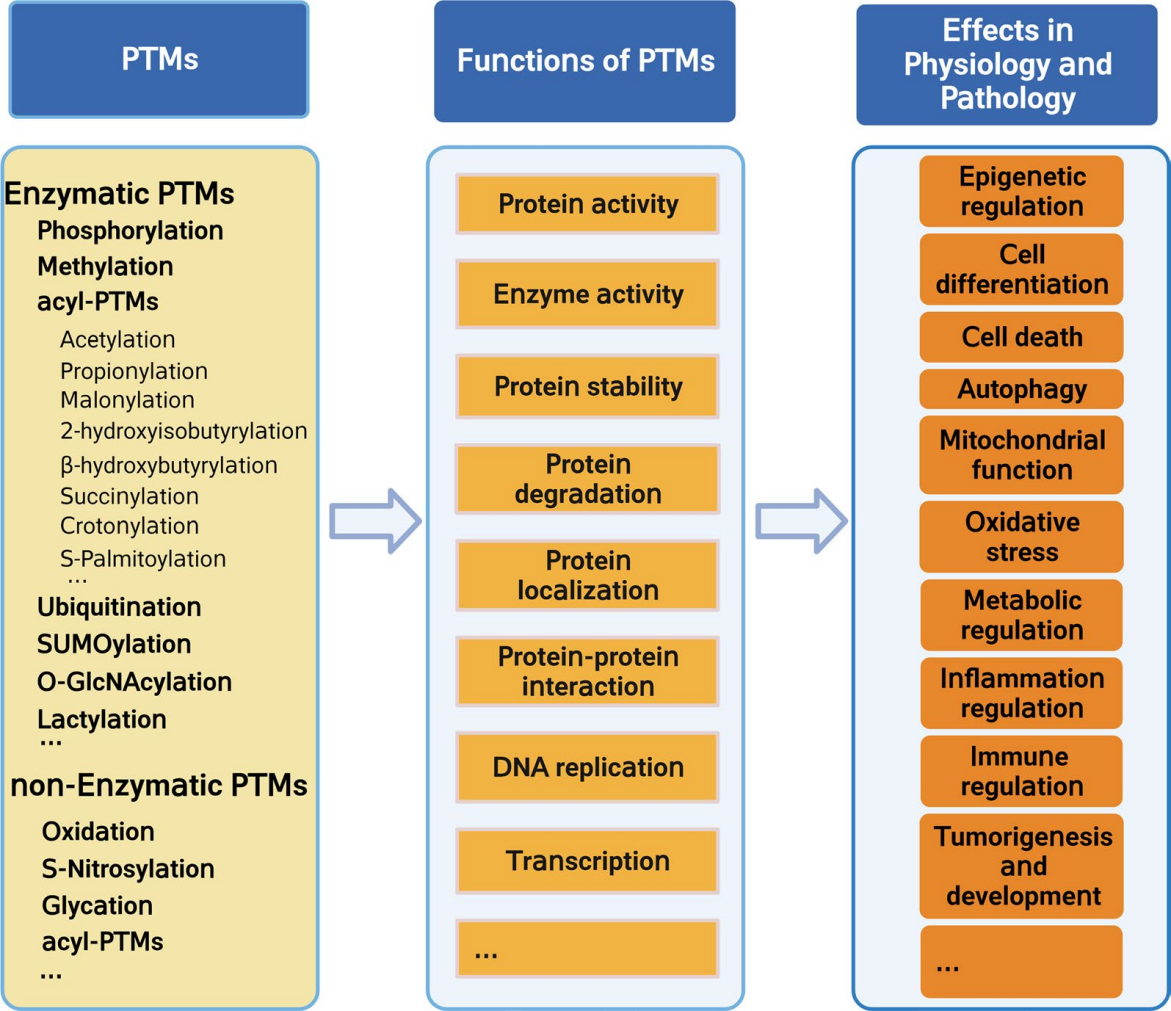
Functions and effect of post-translational modifications in physiology and pathology

PTM refers to the reversible or irreversible covalent processing of some proteins after translation, which occurs at the amino acid side chains, C-terminus, or N-terminus (Ramazi and Zahiri 2021). Based on their biochemical origin, PTMs are divided into enzymatic (ePTMs) and non-enzymatic (nPTMs) (Jennings et al. 2022; Wold 1981). The effects of ePTMs are precisely controlled by PTM enzyme readers, writers, and erasers, which can add or remove modifications (Jennings et al. 2022). In contrast, nPTMs usually occur spontaneously between nucleophilic or redox-sensitive amino acid side chains and reactive metabolites (Harmel and Fiedler 2018). Approximately two-thirds of proteins in vivo undergo PTMs; these modifications include phosphorylation, acetylation, ubiquitination, methylation, and glycosylation. Emerging evidence reveals that PTMs can expand the diversity of proteins by influencing their functions via altering protein structure, localization, and activity. Ultimately, PTMs play a vital role in various physiological and pathophysiological processes, such as cell replication, cell death, transcription regulation, translation regulation, cellular signal transduction, and immune regulation (Fig. 1) (Meng et al. 2021; Patwardhan et al. 2021; Yu et al. 2022), and are also involved in glucose and lipid metabolism. For example, Lorendeau et al. focused on the metabolic regulation of signaling and transcriptional regulation of mammalian target of rapamycin (mTOR), AMP-activated protein kinase (AMPK), and p53, and discussed functional consequences of PTMs on these enzymes (Lorendeau et al. 2015).

In this review, we summarize the types and roles of PTMs and illustrate their molecular mechanisms in regulating glucose and lipid metabolism. Additionally, we highlight the roles and mechanisms of PTMs in diseases associated with aberrant glucose and lipid metabolism. Our review aims to provide insights into the treatment of diseases associated with dysregulated glucose and lipid metabolism.

## Common types of PTMs

PTMs are complex processes that play extremely important roles in almost all cellular activities. Exploring the regulatory processes of PTMs is of great significance for understanding the molecular mechanisms or finding new biomarkers for various diseases. There are currently more than 400 known PTMs. The most common modifications of proteins associated with glucose and lipid metabolism include phosphorylation, acetylation, ubiquitination, SUMOylation, lactylation, methylation, S-glutathionylation, and glycosylation (Table 1).

**Table 1:** Characterizations of several common PTMs

Type of PTMs	Chemical Structure	Modified Amino Acid Residues	Added	Removed	References
Phosphorylation		Ser, Thr, Tyr, His, Asp, Pro, Glu, Cys, Arg, and Lys (Jennings et al. 2022)	Enzymatic (kinases)	Enzymatic (phosphatases)	Jennings et al. (2022)
Ubiquitination		Lys, Cys, Ser, and Thr, (Kelsall 2022)	Enzymatic [ubiquitinactivating (E1), -conjugating (E2), and -ligase (E3)] (Reyes-Turcu et al. 2009)	Enzymatic [deubiquitinating enzymes (DUBs)] (Reyes-Turcu et al. 2009)	Kelsall (2022); Reyes-Turcu et al. (2009)
Methylation		Lys (mono-, di-, and tri-methylation) (Bhat et al. 2021), Arg (mono- and di-methylation of Arg) (Xu and Richard 2021)	Enzymatic (Lys methyltransferase and Protein Arg methyltransferases) (Bhat et al. 2021)	Enzymatic (Lys demethylases, Lys-specific histone demethylase 1and JmjC domain-containing 6) (Bhat et al. 2021; Manni et al. 2022)	Bhat et al. (2021; Xu and Richard (2021)
O-GlcNAcylation		Ser and Thr (Yang and Qian 2017)	Enzymatic (O-GlcNAc transferase) (Wang et al. 2023)	Enzymatic (O-GlcNAcase) (Wang et al. 2023)	Wang et al. (2023); Yang and Qian (2017)
Acetylation		Lys, N-terminus of proteins, thiol group of Cys	Enzymatic (acetyltransferases) and non-enzymatic(S-to-N acyl transfer mechanism)	Enzymatic (deacetylases and sirtuins)	James et al. (2018); Jennings et al. (2022)
SUMOylation		Lys (Chang and Yeh (2020)	Enzymatic [SUMO activating (E1), -conjugating (E2), and -ligase (E3)] (Yeh 2009)	Enzymatic [deSUMOylating enzyme (SENP)] (Yeh 2009)	Chang et al. (2020); Du et al. (2021); Yeh (2009)
Succinylation		Lys	Enzymatic (KAT2A/GCN5) (Wang et al. 2018b) and non-enzymatic	Enzymatic (SIRT5, SIRT7) (Li et al. 2016; Rardin et al. 2013)	Li et al. (2016); Rardin et al. (2013); Wang et al. (2018b)
Lactylation		Lys (Chen et al. 2021a)	Enzymatic [p300/CBP (Zhang et al. 2019b)] and non-enzymatic(lactyl-glutathione) (Chen et al. 2021a)	Unknown	Chen et al. (2021a)
Propionylation		Lys (Tang et al. 2022)	Enzymatic (p300/CBP (Chen et al. 2007), GCN5 (Kebede et al. 2017), PCAF (Kebede et al. 2017), MOF (Han et al. 2018), BRPF1-KAT6 (Yan et al. 2020)) and non-enzymatic (high concentrations of propionyl-CoA) (You et al. 2019)	Enzymatic [SIRT1 (Cheng et al. 2009)]	Chen et al. (2007); Cheng et al. (2009); Han et al. (2018); Kebede et al. (2017); Tang et al. (2022); Yan et al. (2020); You et al. (2019)
Malonylation		Lys (Zou et al. 2023)	Enzymatic (unknown (Zou et al. 2023), donor of malonylation: Malonyl-CoA)	Enzymatic (SIRT5) (Baek et al. 2023; Wang et al. 2022b)	(Baek et al. 2023; Wang et al. (2022b); Zou et al. 2023)
S-Palmitoylation		Cys (Liu et al. 2022b)	Enzymatic (zDHHC palmitoylase family) (Zhou et al. 2023)	Enzymatic (APT1/2, PPT1/ 2, ABHD17A/B/ C) (Zhou et al. 2023)	Liu et al. (2022b); Zhou et al. (2023)
S-Glutathionylation		Cys (Rashdan et al. 2020)	Enzymatic (Glutathione S-transferases (GST) ) and non-enzymatic (Martinez-Ruiz and Lamas 2007; Rashdan et al. 2020)	Enzymatic (Glutaredoxins (Grx), SRX, TRX) (Rashdan et al. 2020)	Martinez-Ruiz and Lamas (2007); Rashdan et al. (2020)
Crotonylation		Lys (Tan et al. 2011), Ser (Liao et al. 2020)	Enzymatic (Crotonyltransferases:p300/CBP (Sabari et al. 2015), KAT8 /MOF (Liu et al. 2017), KAT2B /PCAF (Xu et al. 2017), Gcn5 and Esa1 (Kollenstart et al. 2019))	Enzymatic [HDAC1–3 (Kelly et al. 2018; Madsen and Olsen 2012) and SIRT1-3 (Bao et al. 2014)]	Bao et al. (2014); Kelly et al. (2018); Kollenstart et al. (2019); Liao et al. (2020); Liu et al. (2017); Madsen and Olsen (2012); Sabari et al. (2015); Tan et al. (2011); Wang et al. (2021); Xu et al. (2017)
2-hydroxyisobutyrylation		Lys (Huang et al. 2018a)	Enzymatic [p300 (Huang et al. 2018b) and TIP60 (Wang et al. 2022c)]	Enzymatic [HDAC2 and HDAC3 (Huang et al. 2018a)]	Huang et al. (2018a; b); Wang et al. (2022c)
β-hydroxybutyrylation		Lys (Huang et al. 2021)	Enzymatic [p300/CBP (Huang et al. 2021)]	Enzymatic [HDAC1-3 (Huang et al. 2021), SIRT1-2 (Huang et al. 2021), SIRT3 (Zhang et al. 2019c)]	Huang et al. (2021); Zhang et al. (2019c)

### Phosphorylation

Phosphorylation is the process by which phosphate groups bind to substrates and thus regulate protein activity and interactions under the regulation of protein kinases (Cohen 2002). Phosphorylation affects at least one-third of eukaryotic proteins (Cohen 2000). It is widely involved in regulatory processes, including membrane transport, protein degradation, regulation of enzyme activity (activation or inhibition), and protein interactions. Thus, phosphorylation plays a vital role in regulating cell apoptosis, mitochondrial function, inflammatory response, oxidative stress, cellular signaling, translocation, and autophagy (Carlson et al. 2020; Hepowit et al. 2022; Liu et al. 2021a; Peng et al. 2017; Ross et al. 2023; Zhang et al. 2022). Protein phosphorylation is one of the most common and important PTMs (Sacco et al. 2012). It is a reversible process that is regulated by protein kinases and phosphatases.

The most common phosphorylation sites are in the amino acid side chains of serine (Ser), threonine (Thr), and tyrosine (Tyr) residues (Seok 2021). Overall, phosphorylated Ser is the most abundant (86%), followed by Thr (12%) and Tyr (2%) (Olsen et al. 2006). Phosphorylation plays critical regulatory roles in glycolipid metabolism. For example, phosphorylation of the Ser473 site of protein kinase B (Akt) inhibits the activity of GSK3β, thereby activating glycogen synthase to reduce blood sugar in HepG2 cells (Gao et al. 2018). Additionally, Galectin-3 can mediate cardiac remodeling due to impaired glycolipid metabolism by inhibiting Akt phosphorylation at Thr308/Ser473 (Sun et al. 2021b). AMPK phosphorylation by cellular repressor of E1A stimulated genes 1 (CREG1) can lead to glucose uptake in skeletal muscle cells (Goto et al. 2023). Therefore, phosphorylation sites may have the potential to serve as biomarkers for glucose and lipid metabolism diseases or even as possible therapeutic targets.

### Acetylation

Acetylation, a type of PTM that has been extensively explored, refers to the process of acetyl group transfer from acetyl coenzyme A to lysine or other amino acid residues of target proteins. It is catalyzed by acetyltransferases and regulates gene transcription and signal transduction (Drazic et al. 2016). It is a reversible process that is regulated mainly by lysine acetyltransferases (KATs) and lysine deacetylases. Acetylation is classified as Nα-acetylation, Nε-acetylation, and O-acetylation, depending on the addition of acetyl groups to different amino acids and at different sites (Lee et al. 2010). One of the first modifications of histone discovered was acetylation, wherein an acetyl group is added to lysine residues at the N terminus of histone protein, which regulates gene transcription by affecting the binding of DNA to histones (Verdone et al. 2005). Notably, KAT-mediated histone acetylation affects epigenetic processes (He et al. 2018). For example, the activation of Toll-like receptors can promote histone acetylation and thus regulate Myeloid differentiation primary response 88 (MyD88) and Toll/Interleukin-1 receptor-domain-containing adapter-inducing interferon-β (TRIF) signaling, leading to the activation of adenosine triphosphate (ATP)-citrate lyase and thereby promoting energy metabolism (Lauterbach et al. 2019). Recruitment of hypoxia-inducible factor 1 alpha (HIF1α) to hypoxia-responsive elements induces glucose uptake through its interaction with p300-dependent histone acetylation (Anand et al. 2023). Recently, several non-histone acetylations have been identified that mainly affect gene transcription, DNA damage repair, protein folding, cell division, signal transduction, autophagy (Narita et al. 2019). Moreover, acetylation is important for the regulation of metabolism (Zhao et al. 2010). For instance, Zhang et al. discovered that histone deacetylase 8 could alter the glucose metabolism of hepatocellular carcinoma cells by controlling the acetylation of the PKM2 protein at the K62 site, leading to a predominant utilization of glucose through the pentose phosphate pathway (Zhang et al. 2020a). Thus, the balance between acetylation and deacetylation is crucial, and disruption of this balance may lead to disease development.

### Ubiquitination and SUMOylation

Ubiquitination, a common PTM, refers to the process in which ubiquitin covalently binding to target proteins and is catalyzed by a three-enzyme cascade, composed of E1 ubiquitin-activating enzymes, E2 ubiquitin-conjugating enzymes, and E3 ubiquitin ligases. Ubiquitin (Ub), a highly conserved 76-amino acid protein, contains seven lysine residues (K6, K11, K27, K29, K33, K48, and K63), each of which is ubiquitinated to form distinctive forms of polyubiquitin chains (Swatek and Komender 2016). Various lengths and types of ubiquitinated chains determine the fate of substrate proteins and mediate different signaling pathways. Although ubiquitination mainly regulates the degradation of proteins, studies have found that ubiquitination also plays vital roles in regulating protein activity, protein–protein interactions, subcellular localization, and signal transduction (Komander and Rape 2012; Rajalingam and Dikic 2016). For instance, K48-linked chains are responsible for targeting substrate proteins for proteasomal degradation, while K63-linked chains are involved in several nonproteolytic functions, such as nuclear factor (NF)-κB activation and DNA damage repair (Emmerich et al. 2016; Liu et al. 2018; Yu et al. 2021). Numerous studies have confirmed that ubiquitination is widely involved in various physiological and pathological processes, such as transcriptional regulation, cell proliferation, cell apoptosis, DNA damage repair, and immune regulation (Roberts et al. 2022; Zhong et al. 2022). Notably, ubiquitination is a dynamic and reversible process, and can be counteracted by deubiquitinases (Mevissen and Komander 2017). A dynamic balance between ubiquitination and deubiquitination is necessary to maintain protein homeostasis and function, and abnormalities in the ubiquitin system are associated with the occurrence of many diseases, including neurodegenerative diseases, immune diseases, and cancers (Cockram et al. 2021; Liu et al. 2022a).

SUMOylation is an essential PTM similar to ubiquitination. During SUMOylation, a small ubiquitin-like modifier (SUMO) protein covalently binds to target proteins on lysine residues, which is mediated by a specific SUMO E1 activating enzyme, SUMO E2 conjugating enzyme, and SUMO E3 ligase. Unlike ubiquitination, SUMOylation mainly mediates the localization and functional regulation of target proteins instead of promoting degradation (Vertegaal 2022; Zhao 2018). Notably, SUMOylation is reversible, and deSUMOylation is mediated by SUMO-specific proteases, predominantly of the Sentrin/SUMO-specific proteases (SENPs) family. Imbalances in SUMOylation and deSUMOylation have been observed in the progression of various diseases (Mustfa et al. 2017; Zheng et al. 2020) including metabolism-related diseases (Sadeghi et al. 2023; Sapir 2020; Zhu et al. 2022b). Notably, SUMOylation and deSUMOylation have been found to be important in regulating glucose and lipid metabolism. For example, Zheng et al*.* discovered that adipose lipid storage in mice decreased when SUMO-specific protease 2 (Senp2) was specifically knocked out in adipose tissues (Zheng et al. 2018). Senp2 could regulate adipose lipid storage by suppressing Setdb1 function via the de-SUMOylation of Setdb1, suggesting that Senp2-mediated deSUMOylation regulates lipid metabolism in adipose tissues. In addition, guanosine triphosphate binding protein 4 (GTPBP4) was found to induce dimeric pyruvate kinase M2 SUMOylation and dimer formation through the UBA2-SUMO1 axis, thus promoting aerobic glycolysis in hepatocellular carcinoma (Zhou et al. 2022b). Therefore, further studies on the homeostasis of SUMOylation and deSUMOylation may provide new insights into the diagnosis and treatment of these diseases.

### Glycosylation

Protein glycosylation is one of the most abundant and diverse types of PTMs, in which glycan moieties are added to proteins (Reily et al. 2019). By modulating the structure, stability, and function of proteins, glycosylation plays a profound role in various pathological and physiological processes (Bangarh et al. 2023; Pradeep et al. 2023; Reily et al. 2019). There are two major kinds of protein glycosylation in eukaryotes: N-linked (N-glycosylation) and O-linked (O-glycosylation). N-glycosylation involves the attachment of N-glycans (N-acetylglucosamine/GlcNAc) to the amino group of the Asn residue at the sequence Asn–X–Ser/Thr (where X represents any amino acid except for Pro); it initiates in the endoplasmic reticulum (ER) and then further modifications occur in the Golgi apparatus (Pradeep et al. 2023; Schjoldager et al. 2020). O-glycosylation is more complicated, and refers to the covalent addition of diverse glycans (such as N-acetylgalactosamine (GalNAc), fucose, glucose, xylose, and mannose) to the hydroxyl group of Ser/Thr residues, and also on tyrosine, hydroxylysine, and hydroxyproline; it mostly occurs in the Golgi apparatus (Joshi et al. 2018; Li et al. 2022a, b, c; Schjoldager et al. 2020). Notably, O-linked N-acetylglucosamine modification (O-GlcNAcylation), a unique type of O-glycosylation in which O-linked N-acetylglucosamine (O-GlcNAc) is added to Ser and Thr residues of proteins located in the cytoplasm, nucleus, and mitochondria, has received increasing attention in recent years (Gonzalez-Rellan et al. 2022; Yang and Qian 2017). O-GlcNAcylation is mediated by O-GlcNAc transferase (OGT) and O-GlcNAcase (OGA). OGT catalyzes the addition, whereas OGA reversibly removes protein modifications (Gao et al. 2001; Shafi et al. 2000). Research has indicated that O-GlcNAcylation tunes the functions of protein in various ways, including protein cellular localization, protein stability, and protein–protein interaction (Chang et al. 2020). Interestingly, O-GlcNAcylation and phosphorylation have been shown to participate in extensive crosstalk with each other, as they can both occur on Ser and Thr residues of proteins (Hart et al. 2011). O-GlcNAcylation, which is sensitive to cellular metabolic states, has been proposed to function as a “nutrient and stress sensor” in cells (Bond and Hanover 2013; Ruan et al. 2013). Notably, emerging evidence has shown that glycosylation plays a pivotal role in metabolic diseases. For example, Nishimura et al. discovered that suppression of O-GlcNAcylation in the intestine reduced glucose absorption via inhibiting SGLT1 expression, suggesting that regulating O-GlcNAcylation in the intestine may provide a novel strategy for treating absorption disorders, obesity, and diabetes (Nishimura et al. 2022). In addition, Yung et al*.* revealed that ER stress-mediated perturbation of placental protein glycosylation could lead to the maladaptation of maternal hepatic glucose metabolism, which may be a new mechanism of maternal metabolic disorders (Yung et al. 2023). Thus, considering the intimate relationship between glycosylation and metabolic state, studies targeting the regulatory roles of glycosylation may provide novel insights into the treatment of diseases associated with aberrant metabolism.

### Methylation

Methylation, mediated by methyltransferase, is a widespread phenomenon in both eukaryotes and prokaryotes. The substrates of methylation can be DNA, RNA, and proteins. Among these, protein methylation is a common PTM which occurs in both histone and non-histone proteins (Dai et al. 2021). The most common modification sites of methylation are lysine and arginine residues (Jambhekar et al. 2019). Based on the substrates involved, protein methyltransferases can be divided into categories such as protein lysine methyltransferases (PKMTs) and protein arginine methyltransferases (PRMTs) (Bhat et al. 2021; Dai et al. 2021; Xu and Richard 2021). PKMTs can cause monomethylation, bimethylation, or trimethylation of lysines on their substrates (Bhat et al. 2021). Methylation modifications in arginine include monomethylated arginine, asymmetric dimethylarginine, and symmetric dimethylarginine (Blanc and Richard 2017; Dai et al. 2021). Protein methylation plays an essential role in various intracellular processes, including glucose and lipid metabolism, via regulating the function of target proteins (Biggar and Li 2015; Dilworth et al. 2019; Li et al. 2019; Malecki et al. 2022). For example, Han et al*.* discovered that PRMT6 could mediate asymmetric dimethylation of multiple arginine residues of cAMP-response element binding protein (CREB)-regulated transcriptional coactivator 2 (CRTC2), which enhanced the interaction of CRTC2 with CREB on the promoters of gluconeogenic enzyme-encoding genes and thus played a vital role in hepatic glucose metabolism (Che et al. 2021; Han et al. 2014; Jia et al. 2020). In addition, Jia et al. found that PRMT5 regulates fatty acid metabolism and lipid droplet biogenesis in white adipose tissues, and that Prmt5^AKO^ mice (the Prmt5 gene is specifically present in adipocytes) exhibit sex- and depot-dependent progressive lipodystrophy (Jia et al. 2020). Mechanistically, Prmt5 can not only methylate and release the transcription elongation factor SPT5 from the Berardinelli-Seip congenital lipodystrophy 2 (Bscl2 encodes Seipin, which can mediate lipid droplet biogenesis) promoter but also methylate Sterol Regulatory Element-Binding Transcription Factor 1a (SREBP1a) and promote lipogenic gene expression. Thus, further studies on protein methylation may supply potential therapeutic targets for diseases involving dysregulated glucose and lipid metabolism.

### Other PTMs

In addition to the aforementioned PTMs, other PTMs such as lactylation, methylation, S-glutathionylation, N-glycosylation, and palmitoylation have also been observed to participate in glucose and lipid metabolism-related diseases. S-glutathionylation is the formation of mixed disulfides between glutathione and cysteine residues in proteins, which can lead to enhanced or suppressed protein activity (Dalle-Donne et al. 2009). A study performed by Dong et al*.* found that S-glutathionylation of the AMPK-α catalytic subunit could activate AMPK to improve glucose transportation and degradation while inhibiting glycogen synthesis and maintaining redox balance under a low reactive oxygen species microenvironment, providing new insights into diabetes treatment (Dong et al. 2016). However, many other PTMs have not been studied for their role in glucose and lipid metabolism. Given the extensive regulatory roles of PTMs in protein function, future studies should investigate the regulatory roles and mechanisms of various PTMs in glucose and lipid metabolism to provide potential targets for treatment and diagnosis.

In conclusion, PTMs play important roles in protein functions and participate in various biological processes. Exploring their regulatory roles in glucose and lipid metabolism may provide the basis for clinical diagnosis and therapy.

## Roles of PTMs in glucose and lipid metabolism

### Roles of PTMs in glucose metabolism

**Fig. 2:**
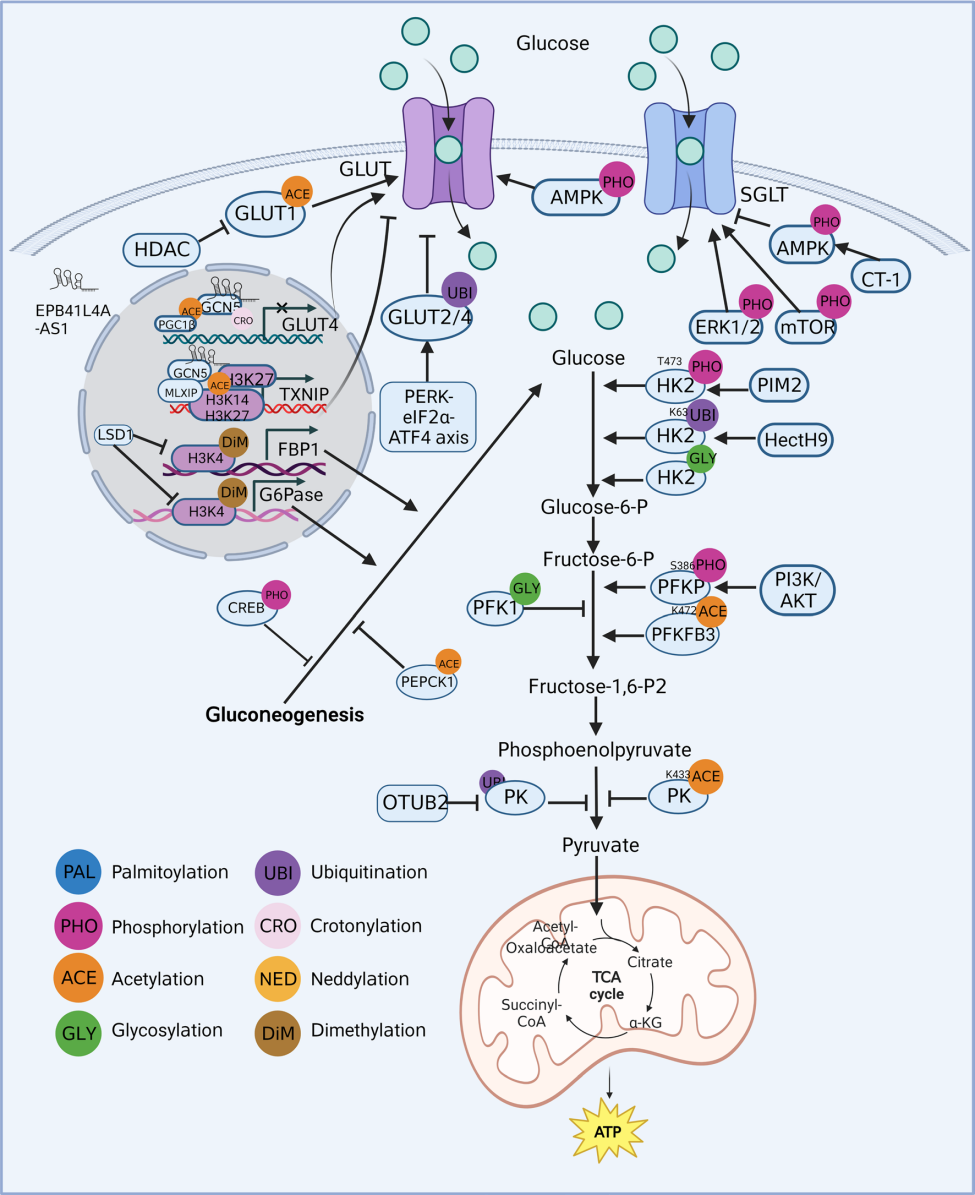
Roles of post-translational modifications in glucose metabolism

Cell cycle, growth, apoptosis, and energy metabolism are critically affected by glucose metabolism (Mulukutla et al. 2010). Glycolysis (involving three irreversible reactions) and gluconeogenesis (involving four irreversible reactions) are the central processes of glucose metabolism (Chandel 2021). Disorders in glucose metabolism primarily involve disruptions in energy and substance metabolism, and they participate in various pathological processes. For example, dysregulated glucose metabolism is involved in diabetes mellitus and AD (Cao et al. 2022; Huang et al. 2023). The reprogrammed glucose metabolism in the enhanced Warburg effect (or aerobic glycolysis) is considered a hallmark of cancer (Povero 2023). A better understanding of the regulation and molecular mechanisms involved in glucose metabolism can help us to understand the basis of many metabolic disorders. Recent studies have found that rate-limiting enzymes in glucose metabolism, such as facilitated-diffusion glucose transporters (GLUT), phosphofructokinase (PFK), and phosphoenolpyruvate carboxykinase (PEPCK), are tightly regulated by several PTMs, including phosphorylation, acetylation, ubiquitination, glycosylation, crotonylation, and dimethylation (Fig. 2) (Ahmed et al. 2023; He et al. 2022; Yi et al. 2012). For example, glycosylation inhibits PFK1 activity and redirects the flux of glucose from glycolysis through the pentose phosphate pathway (Yi et al. 2012). PEPCK is an important enzyme in the gluconeogenic pathway that catalyzes the conversion of oxaloacetate to phosphoenolpyruvate, thereby participating in glucose synthesis. For example, phosphorylation of both AMPK and forkhead box transcription factor O1 (FoxO1) results in a downregulation of PEPCK and G6Pase, thereby promoting glucose uptake and inhibiting glucose production (Ahmed et al. 2023). PEPCK acetylation, which can occur at various amino acid residues, is emerging as an important regulatory mechanism of its activity and is linked to metabolic diseases (Marin-Hernandez et al. 2022; Xiong et al. 2011; Zhang et al. 2018). In addition to PTMs of PEPCK, other rate-limiting enzymes have also been shown to play important roles. LINC00930 can recruit the retinoblastoma binding protein 5 and general control nonderepressible 5 complex to the promoter of PFKFB3, increasing H3K4 trimethylation and H3K9 acetylation levels and transactivating PFKFB3, thereby promoting glycolytic flux (He et al. 2022). In addition, we found that crotonylation and dimethylation are also involved in glucose metabolism. Crotonylation is a type of acylation that regulates gene expression and metabolic homeostasis, and a recent study has shown its involvement in the regulation of energy metabolism (Gowans et al. 2019). Dimethylation is a process that adds two methyl groups to proteins and is known to regulate gene expression (Jackson et al. 2004); it can also affect glucose metabolism (Pan et al. 2013).

#### PTMs in glucose transport

The initial and limiting step in glucose metabolism is glucose transport through the cell membrane via glucose transport proteins. There are two families of cellular glucose transporters: GLUT and sodium-dependent glucose transporters (SGLTs) (Navale and Paranjape 2016). Studies have shown that PTMs regulate GLUT and SGLT in glucose transport. PTMs directly reflect GLUT activity. For example, GLUT 1 and 4 are upregulated following histone deacetylase inhibition, accompanied by an increase in GLUT1 acetylation (Chen et al. 2015a). ER stress-mediated ubiquitination of GLUT-2 and GLUT-4 during hyperglycemia reduces glucose uptake in the liver, exacerbating diabetic pathophysiology (Kumar et al. 2022). Moreover, PTMs indirectly reflect GLUT levels. For instance, phosphorylation of AMPK increases glucose uptake in myocytes for ATP production by mediating the expression and translocation of GLUT-4 protein (Zhang et al. 2019a). Additionally, Liao et al. found that the lncRNA EPB41L4A-AS1 increases histone H3K27 crotonylation in the GLUT-4 promoter region and non-histone PGC1-β acetylation, which inhibits GLUT-4 transcription and suppresses glucose uptake in muscle cells (Liao et al. 2022). Insulin promotes AKT phosphorylation and thus increases GLUT-1 at the plasma membrane in adipocytes to facilitate glucose uptake (Shimamoto et al. 2019). Thioredoxin Interacting Protein (TXNIP) also participates in glucose transport. It is a negative regulator of cellular glucose uptake, reducing glucose influx by promoting GLUT1 endocytosis. It also serves as a direct substrate of AKT, mediating AKT-dependent acute glucose influx, and functions as an adaptor for basal endocytosis of GLUT4 in vivo (Waldhart et al. 2017). SGLTs are also regulated by PTMs. For instance, inhibition of extracellular signal-regulated protein kinase (ERK1/2) and mTOR phosphorylation reduces SGLT-1-mediated glucose uptake (Di Franco et al. 2017). Furthermore, Cardiotrophin-1 inhibits intestinal sugar absorption by reducing SGLT-1 levels through AMPK (Lopez-Yoldi et al. 2016). Overall, PTMs regulate GLUT and SGLT via direct and indirect mechanisms that affect glucose transport. These findings demonstrate that PTMs, such as phosphorylation and acetylation, participate in glucose transport. GLUT and SGLT can serve as interesting therapeutic targets for combating abnormal glucose metabolism-related diseases.

#### PTMs in glycolysis

Glycolysis is the first step in the breakdown of glucose to produce high-energy molecules ATP and NADH. This process rapidly generates energy by breaking down glucose into pyruvate in the cytosol (Baker and Rutter 2023). Three crucial rate-limiting enzymes, hexokinase (HK), phosphofructokinase (PFK), and pyruvate kinase (PK), control the flux of glycolysis. The activity and protein content of rate-limiting enzymes have essential effects on glucose metabolism. Investigation of the regulatory mechanisms may provide novel insights into therapies for diseases associated with dysregulated glucose metabolism.

Numerous studies have shown that PTMs regulate glycolytic processes by regulating the translocation, content, and stability of rate-limiting enzymes. Yang et al*.* found that phosphorylation of hexokinase 2 (HK2) (T473) increased its activity, ultimately enhancing glucose consumption and lactate production (Yang et al. 2018). HectH9-mediated K63-linked ubiquitination is selective for HK2 regulation, and HectH9 works through HK2 to regulate glycolysis (Lee et al. 2019). Baldini et al. found that O-GlcNAc cycling in HK in hepatocytes is a novel way to regulate HK expression and increase glucose entry into liver cells (Baldini et al. 2016), supporting the crucial roles of PTMs in the glycolytic process. In addition, PTMs play a vital role in glycolysis by regulating PFK. Jeon et al. found that phosphorylation of PFKP (S386) mediated by PI3K/AKT could promote the Warburg effect (Jeon et al. 2021). The Warburg effect is characterized by increased glycolysis and lactate production regardless of oxygen availability (Vander et al. 2009). Furthermore, Li et al. found that acetylation of PFKFB3 (K472) impaired the activity of the nuclear localization signal and resulted in PFKFB3 accumulation in the cytoplasm, leading to PFKFB3 activation and enhanced glycolysis (Li et al. 2018a). O-GlcNAcylation of PFK1 (S529) inhibits its activity and regulates the glycolytic pathway through the pentose phosphate pathway (Yi et al. 2012). Moreover, PTMs regulate the final step of glycolysis by influencing the oligomeric state, subcellular localization, and biological activity of PKs. For instance, phosphorylation of pyruvate kinase muscle isozyme M2 (PKM2) (Y105) has been suggested to facilitate the Warburg effect and tumor cell growth (Kalaiarasan et al. 2014). Furthermore, acetylation of PKM2 (K433) was associated with the degradation of PKM2 and decreased PK activity (Jin et al. 2020). Wang et al. found that ubiquitin aldehyde binding 2 regulates PKM2 stability and nuclear repositioning by inhibiting its ubiquitination and blocking the interaction between PKM2 and its ubiquitin E3 ligase, thereby enhancing PKM2 activity and promoting glycolysis (Wang et al. 2022a). Chaiyawat et al. found that lower O-GlcNAcylation levels led to decreased PKM2 expression but induced higher PKM2-specific activity (Chaiyawat et al. 2015). In glycolysis, carbohydrate response element binding protein (ChREBP) binds to the promoter of liver-type pyruvate kinase and promotes the conversion of phosphoenolpyruvate to PK (Uyeda and Repa 2006). ChREBP is primarily expressed in the liver and adipose tissue and is responsible for the transcriptional control of genes involved in glucose utilization and storage (Ortega-Prieto et al. 2019). The regulation of ChREBP is complex and involves various PTMs, including phosphorylation. Under conditions of high glucose availability, ChREBP is phosphorylated by PKA in the cytoplasm. This phosphorylation event prevents ChREBP from entering the nucleus, and inhibits its transcriptional activity (Davies et al. 2008). However, under conditions of low glucose levels or increased cellular energy demand, ChREBP is dephosphorylated, allowing it to translocate into the nucleus and activate the transcription of target genes involved in glucose metabolism (Nakagawa et al. 2013). The phosphorylation status of ChREBP is tightly controlled by cellular glucose levels and energy status, allowing for fine-tuning of glucose homeostasis in response to changing metabolic demands. Regulation of glycolysis is not only exerted by the expression of glycolytic genes and interactions of glycolytic proteins within their environment but also by PTMs and transcriptional regulation. Various PTMs participate in glycolysis by regulating protein functions and may serve as significant therapeutic targets in diseases involving abnormal glucose metabolism.

#### PTMs in gluconeogenesis

Gluconeogenesis is a process by which non-carbohydrate precursor molecules are converted to glucose. There are two mechanisms that regulate gluconeogenesis metabolic pathways: direct regulation through rate-limiting enzymes and indirect regulation through non-rate-limiting enzymes. The key enzymes involved in regulating the rate of gluconeogenesis include PEPCK, glucose 6-phosphatase (G6Pase), pyruvate carboxylase (PC), and fructose-1,6-bisphosphatase (FBP-1). PTMs regulate gluconeogenesis by controlling enzyme activity. For example, PEPCK acetylation stimulates its interaction with E3 ubiquitin ligase (Ubiquitin Protein Ligase E3 Component N-Recognin 5) leading to PEPCK degradation in a proteasome-dependent manner (Jiang et al. 2011). Moreover, an increased level of acetylation and a decreased level of ubiquitination in PEPCK protein in mouse hepatocytes blocks PEPCK protein degradation and enhances hepatic glucose production (Wang et al. 2022d). Additionally, increased gluconeogenesis and decreased intracellular glycogen content result from increased H3K4 dimethylation at the G6Pase promoter (Pan et al. 2013). However, in a previous study, it was found that during gluconeogenesis, even though PEPCK expression was reduced by 90% in the liver after the targeted deletion of the *PEPCK* gene in mice, there was only a 40% reduction in gluconeogenic flux (Johanns et al. 2016). This indicates that the regulation of non-rate-limiting enzymes by PTMs also greatly influences glucose metabolism, as demonstrated in other studies (Gonzalez-Rellan et al. 2022; He et al. 2023; Li et al. 2023; Sun et al. 2020). For example, glucose starvation decreases histone acetylation at multiple sites on H3 (K9, K18, K23, and K27) to activate gluconeogenic and fat metabolism genes (Hsieh et al. 2022). Moreover, phosphorylation of CREB-regulated transcription coactivator 2 and histone deacetylase 5 by AMPK inhibits glucose production (Hunter et al. 2018). Overall, through direct or indirect effects, various PTMs play a vital role in glyconeogenesis, and may be a potential target for its regulation.

In summary, PTMs regulate glucose metabolism, including glucose transport, glycolysis, and gluconeogenesis. They also regulate the activity and protein content of enzymes in these metabolic pathways, which in turn affect glucose metabolism. Therefore, investigating the regulatory mechanisms of PTMs may provide new insights into the development of therapies for diseases associated with dysregulated glucose metabolism.

### Roles of PTMs in lipid metabolism

**Fig. 3:**
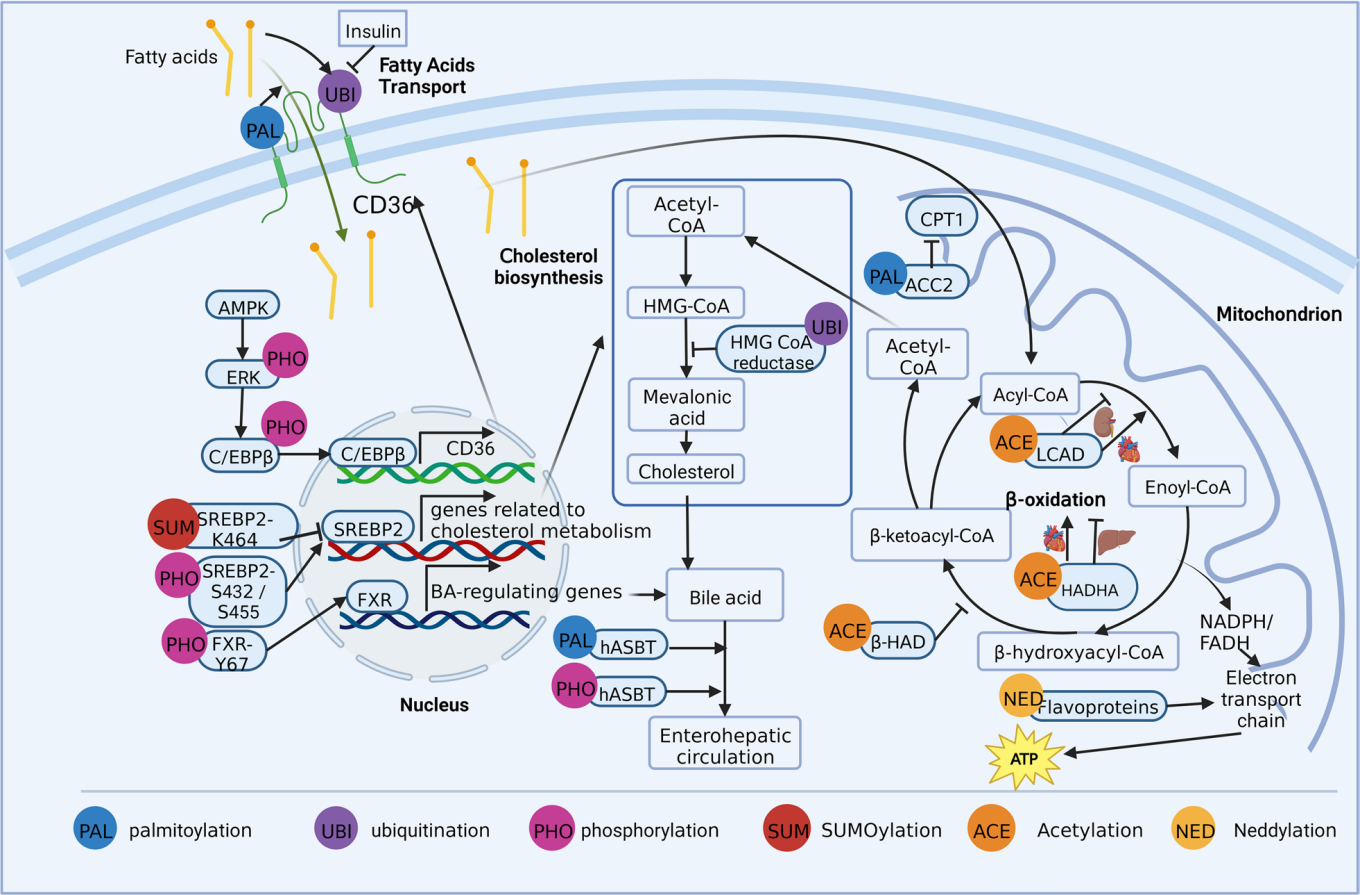
Roles of post-translational modifications in lipid metabolism

Lipids are hydrophobic molecules that include triacylglycerol, cholesterol, cholesterol esters, phospholipids, glycolipids, and lipoproteins. Lipid metabolism influences various biological processes, including energy metabolism, signal transduction, and the biosynthesis of membrane lipids (Bian et al. 2021; Marx 2022). Aberrant lipid metabolism is closely related to many diseases. For example, insufficient fatty acid uptake and utilization lead to malnutrition, whereas excessive lipid storage and hyperlipidemia are involved in atherosclerosis, obesity, and non-alcoholic fatty liver disease (Liu et al. 2021b; Lu et al. 2021). A better understanding of the factors that regulate lipid metabolism may provide new potential therapeutic strategies. Notably, a growing body of research has found that PTMs play vital roles in lipid metabolism by affecting key proteins at pivotal steps (Fig. 3).

#### PTMs in fatty acid transport

When the body requires energy, triglycerides stored in the adipose tissue are mobilized and decomposed into free fatty acids and glycerol, which are released into the blood and transported to tissues requiring energy. This process involves various transporters, including fatty acid translocase (FAT, also named cluster of differentiation 36, CD36), fatty acid transport proteins (FATP), and fatty acid-binding protein (FABP) (Li et al. 2022a). CD36, a key mediator of lipid transport, facilitates the transport and uptake of long-chain fatty acids (Li et al. 2022c). Several PTMs have been shown to affect CD36-mediated lipid transport (Luiken et al. 2016). For instance, Zeng et al. discovered that inhibition of CD36 palmitoylation could increase its localization to mitochondria and enhance its interaction with long-chain acyl-CoA synthetase 1, ultimately enhancing hepatic fatty acid β-oxidation (Zeng et al. 2022). In addition, ubiquitination regulates CD36 levels. However, Smith et al. discovered that fatty acids could strongly enhance the ubiquitination of CD36 and reduce CD36 protein levels, whereas insulin could reduce CD36 ubiquitination and increase CD36 protein levels (Smith et al. 2008). This opposing regulation of CD36 may modulate fatty acid uptake (Smith et al. 2008). Notably, PTMs can also affect CD36 indirectly by modifying upstream kinases and transcription factors. Choi et al. demonstrated that phosphorylation of AMPK by berberine could induce the phosphorylation of ERK1/2 and subsequently cause CCAAT/enhancer-binding protein β (C/EBPβ) binding to the C/EBP-response element in the CD36 promoter, ultimately leading to increased CD36 expression in hepatocytes (Choi et al. 2017).

Studies on PTMs of FATP and FABP are limited. Insulin receptor is a receptor tyrosine kinase. Nielsen et al*.* discovered that in myocytes and mammary epithelial cells, FABP was phosphorylated in response to insulin stimulation in the presence of tyrosine phosphatase inhibitors, indicating that these phosphorylated FABPs might serve as an intermediary in signal transduction pathways between the insulin receptors and lipid metabolism (Nielsen et al. 1994, Nielsen and Spener 1993). However, no phosphorylation was found in FABP from rat soleus muscle (M-FABP) upon insulin stimulation, suggesting that tyrosine phosphorylation of M-FABP was not an important physiological phenomenon (Prinsen et al. 1994). Thus, the effects of phosphorylation on FABPs from different tissues might be diverse and require further study. Further research is also needed on the role of other PTMs in FABP, and in various PTMs in FATP.

In summary, several PTMs can affect fatty acid transport by modulating the key transporter protein, CD36. Nevertheless, further research is required to explore the effects of other PTMs on CD36 and various PTMs on FATP and FABP to provide novel insights into the regulation of fatty acid transport.

#### PTMs in fatty acid oxidation (FAO)

Fatty acids, via their oxidation, serve as the primary energy sources in humans and mammals. Normal FAO is essential in maintaining many biological processes, whereas dysregulated FAO is associated with many diseases. FAO is a complex process and can be modulated by many mechanisms. Accumulating evidence has shown that PTMs play vital roles in FAO.

Long-chain acyl-CoA dehydrogenases (LCAD), β-hydroxyacyl-CoA dehydrogenase (β-HAD), hydroxyacyl-CoA dehydrogenase trifunctional multienzyme complex subunit α (HADHA), and acetyl-CoA carboxylase 2 (ACC2) are key FAO enzymes, and PTMs have been known to regulate their activity. GCN5L1 is an acetylase that counteracts the deacetylation function of SIRT3 (Lv et al. 2019). Lv et al. reported that LCAD and β-HAD under the control of GCN5 general control of amino acid synthesis 5-like 1 (GCN5L1) led to decreased enzymatic activity and impaired FAO rate in a dyslipidemia-induced kidney injury model. Similarly, Thapa et al. demonstrated that acetylation of HADHA by GCN5L1 decreased its activity in HepG2 cells (Thapa et al. 2018). However, another study (Thapa et al. 2017) reported opposing results in the heart; acetylation of LCAD and HADHA by GCN5L1 enhanced FAO. These discrepancies may be caused by differences in the tissues controlling the acetylation status and FAO or tissue-specific acetylated sites of the enzymes, which should be explored in future studies. Acetyl-CoA carboxylase (ACC) is involved in FAO by inhibiting a key rate-limiting enzyme, carnitine palmitoyl transferase (CPT-I), through malonyl-CoA. A study conducted by O’Neill et al. using ACC2 S212A knock-in mice found that phosphorylation of ACC2 at S221 (S212 in mice) by AMPK regulates skeletal muscle FAO and insulin sensitivity (O’Neill et al. 2014). Neddylation is a ubiquitin-like PTM, in which the ubiquitin-like protein neural precursor cell expressed, developmentally downregulated protein 8 (NEDD8) binds to the target protein by three enzymes: the activating enzyme, conjugating enzyme, and ligase (Zhu et al. 2022a). By affecting the stability, conformation, subcellular localization, and activity of target proteins, neddylation plays critical roles in diverse biological processes including metabolism, immunity, and tumorigenesis (Zou and Zhang 2021). Zhang et al. found that hepatic neddylation could stabilize flavoproteins, thus promoting FAO in neonatal mouse livers and preventing fasting-induced steatosis in adult mice (Zhang et al. 2020b). Flavoproteins are components of the electron transport chain in the mitochondria and are essential for energy metabolism.

In summary, various PTMs regulate FAO, which may provide an extremely promising insight for treating diseases associated with dysregulated FAO. However, it is notable that the function of a type of PTM may vary when it acts on different tissues or modifies different sites of the same protein. Thus, in the future, experimental studies are needed to explore the roles and mechanisms of various PTMs in regulating genes related to FAO in different sites, tissues, or organs, which may provide a basis for developing precise treatments.

#### PTMs in cholesterol metabolism

Cholesterol and cholesterol esters determine the composition of the plasma membrane, act as precursors of steroid hormones and bile acids, and regulate various cellular functions (Duan et al. 2022). However, excess cholesterol is harmful and can lead to many diseases, such as cardiovascular diseases. For instance, defects in cholesterol biosynthesis cause Smith–Lemli–Opitz syndrome, a neurological and developmental disorder characterized by multiple developmental defects (Tomita et al. 2022). Excessive cholesterol levels are also associated with atherosclerosis (Baumer et al. 2020). Thus, the balance between cholesterol biosynthesis, uptake, transport, and secretion is of great importance for maintaining cholesterol homeostasis. Notably, emerging evidence has indicated that PTMs are vital in regulating cholesterol metabolism.

Several key enzymes and proteins involved in cholesterol metabolism are reportedly regulated by PTMs (Byun et al. 2018; Johnson and DeBose-Boyd 2018; Shimano and Sato 2017). For example, 3-hydroxy-3-methylglutaryl coenzyme A reductase (HMG-CoA reductase) can be ubiquitinated and then degraded through endoplasmic reticulum-associated degradation (Johnson and DeBose-Boyd 2018). HMG-CoA reductase, which catalyzes the synthesis of mevalonic acid, is the rate-limiting enzyme in cholesterol biosynthesis. The regulation of its levels may lead to elevated levels of cholesterol and its precursors, inhibiting cholesterol synthesis and regulating cholesterol homeostasis (Johnson and DeBose-Boyd 2018). PTMs can also participate in cholesterol metabolism by regulating the activity of sterol regulatory element binding protein (SREBP)-2, an isoform of the transcription factor family SREBP, which is involved in regulating the transcription of genes related to cholesterol metabolism (Shimano and Sato 2017). The transactivation capacity of SREBP-2 was shown to decrease when it was modified by SUMO-1 at Lys464 (Hirano et al. 2003). In contrast, phosphorylation of SREBP-2 at Ser-432 and Ser-455 reportedly increased its transactivation capacity (Kotzka et al. 2004). These results indicate that the activity of SREBP-2 is commonly regulated by PTMs, which could provide novel insights into maintaining cholesterol metabolism. Notably, PTMs are also involved in the regulation of bile acid metabolism, which is the main pathway for cholesterol utilization. The Farnesoid X receptor (FXR), which transcriptionally regulates genes involved in bile acid metabolism, is essential in maintaining bile acid homeostasis. Byun et al. discovered that, in response to postprandial FGF19, phosphorylation of FXR by Src was critical for its transcriptional regulation of bile acid levels and may be a potential therapeutic target for treating bile acid-related diseases (Byun et al. 2018). In addition, phosphorylation and palmitoylation of the human apical sodium-dependent bile acid transporter (hASBT), responsible for the reclamation of bile acids from the intestinal lumen, reportedly regulate membrane expression, function, and stability of hASBT, ultimately influencing bile acid enterohepatic circulation and metabolism (Ayewoh et al. 2021; Chothe et al. 2019). Taken together, PTMs, by influencing the expression and activity of key enzymes and transcription factors, may participate in cholesterol metabolism. Considering the importance of cholesterol metabolism balance in maintaining normal physiological processes, these results may provide novel insights for the treatment of diseases caused by dysregulated cholesterol metabolism.

In conclusion, PTMs influence various processes of lipid metabolism via the modification of key proteins. PTMs also influence lipid storage (Qian et al. 2017) and adipogenesis (Su et al. 2022). Considering their importance in regulating target protein expression, activity, and location, exploring the roles of various PTMs in lipid metabolism may lead to the treatment of diseases caused by dysregulated lipid metabolism. However, since the same modification at various sites or various modifications at the same site may lead to different effects on the target protein, the detailed roles of PTMs require further investigation for precise clinical treatments.

## Roles of PTMs in diseases associated with dysregulated glucose and lipid metabolism

**Fig. 4:**
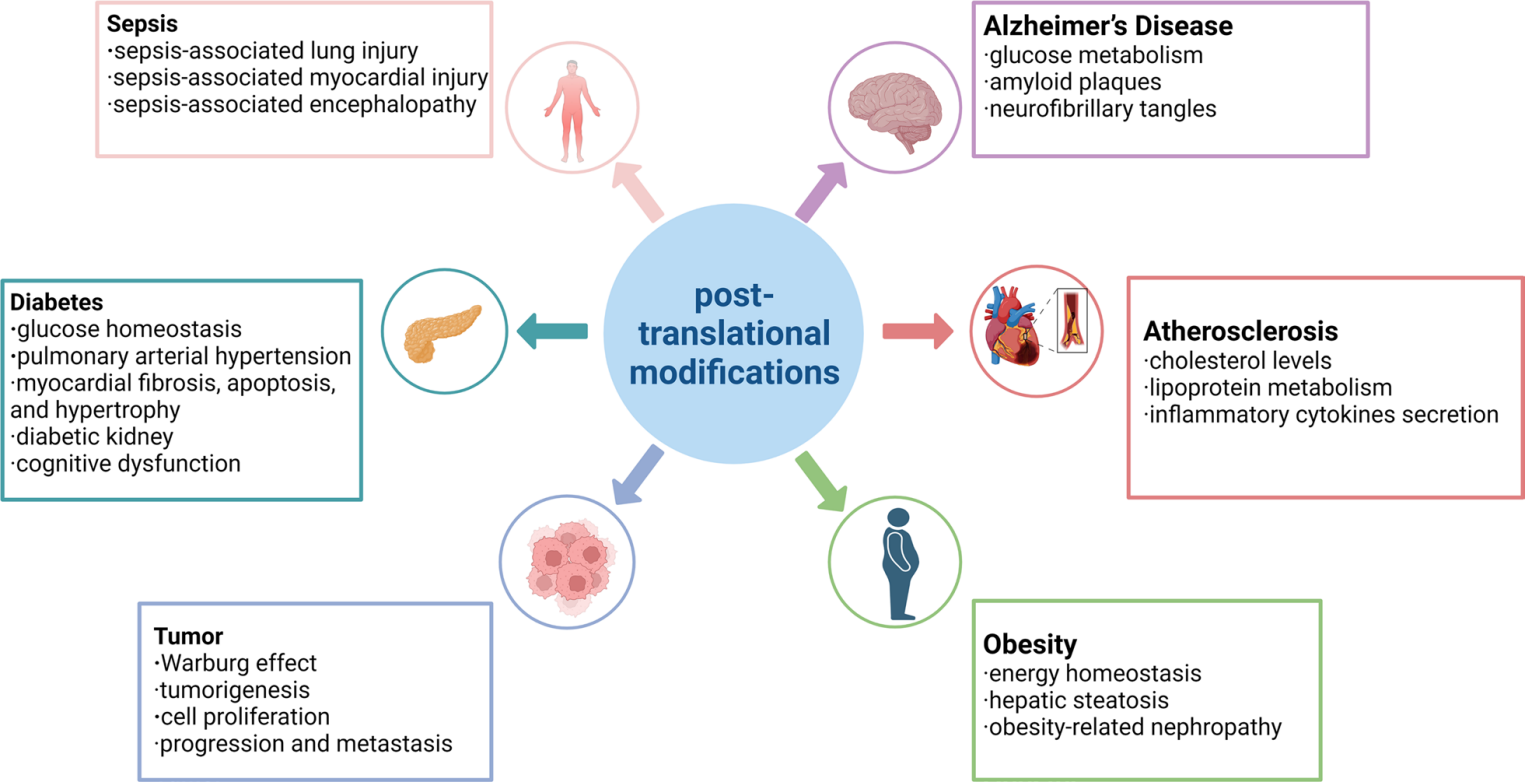
Roles of PTMs in metabolic diseases

Dysregulated glucose and lipid metabolism are associated with several acute and chronic metabolic diseases, including diabetes mellitus, Alzheimer’s disease, atherosclerosis (AS), obesity, tumor and sepsis. Recently, an increasing number of studies have shown that PTMs play a vital role in these metabolic diseases by regulating glucose and lipid metabolism (Fig. 4).

### Role of PTMs in diabetes mellitus (DM)

Diabetes mellitus (DM) is a chronic metabolic disorder characterized by elevated blood glucose levels resulting from inadequate insulin production. Diabetes can be differentiated into type 1 diabetes mellitus (T1DM) and type 2 diabetes mellitus (T2DM). T1DM is an autoimmune disease resulting from the loss of immune tolerance to beta cell autoantigens. PTMs have been shown to regulate glucose and lipid metabolism and are associated with the pathology of DM; for instance, higher doses of AM-879 inhibit Ser273 phosphorylation to improve insulin sensitivity and glucose disappearance rates (Terra et al. 2023). Moreover, glucose and lipid metabolism disturbances promote myocardial fibrosis, apoptosis, and hypertrophy by inhibiting phosphorylation of Akt (T308 or S473) mediated by galectin-3 (Sun et al. 2021b). Although the expression level of enoyl-CoA-hydratase/3-hydroxyacyl-CoA dehydrogenase decreases, acetylation enhances its activity, thereby overall increasing β-oxidation processes in the kidneys of diabetic individuals (Sas et al. 2016). *Artemisia dracunculus* L. extract increased the phosphorylation of AMPK (T172), affecting downstream signaling of AMPK, inhibiting ACC and increasing SIRT1 protein levels to improve glucose homeostasis by enhancing insulin action and reducing ectopic lipid accumulation (Vandanmagsar et al. 2021). Recent studies have shown that PTMs are also involved in glucose transport, affecting the development of diabetes. For instance, the inhibition of SGLT1 expression and lack of O-GlcNAcylation in the gut decreased glucose absorption (Nishimura et al. 2022). Yang et al. found that *Epigynum auritum* increased phosphorylation levels of Akt, AMPK, and GSK-3β, which in turn upregulated the expression of GLUT-2 and GLUT-4, thus exerting a hypoglycemic effect (Yang et al. 2022a). Increased O-GlcNAc levels in diabetes decrease ERα activity, which reduces the brain's ability to utilize glucose, reduces the release of neurotrophic factors, and increases the risk of neuronal oxidative stress (Shi et al. 2021). Furthermore, the phosphorylation of ribosomal protein S6 kinase and the SREBP1 pathway in nearby hepatocytes is influenced by calpain proteolysis in cultured ECs, leading to the induction of de novo lipogenesis (Akasu et al. 2022). The identification and characterization of these PTMs present significant challenges and research on PTMs can provide substantial insights into the biological functions of these proteins.

### Role of PTMs in Alzheimer’s disease (AD)

AD, a neurodegenerative disease associated with decreased cognitive abilities, is characterized by dysregulated brain glucose metabolism and the accumulation of abnormal protein deposits called myloid plaques and neurofibrillary tangles (NFTs) (Guillozet et al. 2003). PTMs such as phosphorylation, O-GlcNAcylation, and succinylation play a vital role in AD pathogenesis. Tau, a cytosolic phosphoprotein associated with microtubule assembly, is modified by various PTMs. Hyperphosphorylated tau is a major component of NFTs in AD; this indicates that inhibiting the hyperphosphorylation of tau may be a novel therapeutic target for AD. For instance, Zhou et al*.* discovered that Sirt2 was involved in tau phosphorylation through ERK activation in vivo and in vitro, providing novel insights for the treatment of AD (Zhou et al. 2022a). Xu et al. demonstrated that electroacupuncture preserves cognition in an AD mouse model. At the molecular level, electroacupuncture enhances glucose metabolism and inhibits abnormal phosphorylation of tau protein via the AKT/GSK3β signaling pathway (Xu et al. 2020). Regarding O-GlcNAcylation, Liu et al. revealed that downregulation of tau O-GlcNAcylation leads to abnormal hyperphosphorylation of tau and neurofibrillary degeneration in AD (Liu et al. 2004, 2009). Using proteomic analysis, Tramutola et al. discovered that proteins with reduced O-GlcNAcylation levels are involved in key pathways in the progression of AD, such as neuronal structure, protein degradation, and glucose metabolism (Tramutola et al. 2018). Pinho et al. found that globally reduced O-GlcNAcylation levels were associated with impaired mitochondrial bioenergetic function, disruption of the mitochondrial network, and loss of cell viability in in vitro models of AD (Pinho et al. 2019). These results provide a better understanding of the role of O-GlcNAcylation in AD. In recent years, other PTMs, such as succinylation, lactylation, glycosylation, palmitoylation, and nitrosylation have also been found to participate in AD (Abrams et al. 2011; Andrew et al. 2017; Bukke et al. 2020; Pan et al. 2022; Yang et al. 2022b). Yang et al. discovered that succinylation of the amyloid precursor protein promoted amyloid plaque formation, and succinylation of tau promoted its aggregation to NFTs, indicating that succinylation may be associated with AD (Yang et al. 2022b). Moreover, dysregulated O-GlcNAcylation and succinylation in AD may be caused by abnormalities in brain glucose metabolism (Liu et al. 2009; Yang et al. 2022b), suggesting that these PTMs may link dysregulated brain glucose metabolism to pathological alterations in AD. In conclusion, various PTMs regulate the pathogenesis of AD and provide insights into potential therapeutic targets for AD.

### Role of PTMs in atherosclerosis (AS)

AS is characterized by large and medium arteries, which are caused by metabolic disorders of the arterial vessel wall, and is commonly considered a major contributor to cardiovascular diseases (CVDs), including stroke and myocardial infarction (Meng et al. 2022). PTMs have been associated with the pathology of AS by regulating glucose and lipid metabolism. First, abnormal levels of protein phosphorylation have been found to be closely related to the occurrence and development of AS. FGF19-induced phosphorylation of hepatic FXR is a nuclear receptor that plays an important role in maintaining metabolic homeostasis via the transcriptional control of many genes. Byun et al*.* reported that FXR could maintain cholesterol levels and thus protect against AS (Byun et al. 2019).

Glycosylation has also been shown to participate in regulating AS. Altered glycosylation of various proteins involved in lipoprotein metabolism, such as apolipoproteins and lipoprotein receptors, can change their expression and/or function, thus affecting AS development (Pirillo et al. 2021). For example, low density lipoprotein receptor (LDLR), a glycoprotein, regulates circulating LDL-C levels by binding to LDLs. Glycosylation of LDLR is essential for its function by maintaining its expression and binding affinity with LDLs and very-low-density lipoproteins (Filipovic 1989; van den Boogert et al. 2019; Wang et al. 2018a). Ye et al*.* showed that the expression of GalNAc-T4 (GALNT4) and protein O-glycosylation were both increased in plaques in ApoE^−^/^−^mice, and GALNT4 could increase O-glycosylation of PSGL-1 via the Akt/mTOR and NF-κB pathways, thus priming adhesion and transmigration of monocytes in AS. These results provide novel insights into the role of O-glycosylation in the pathogenesis of AS, suggesting that GALNT4 may be a potential target for AS treatment (Ye et al. 2022).

Moreover, S-nitrosylation and SUMOylation were found to play important roles in AS (Chen et al. 2015b; Li et al. 2018b; Liu et al. 2020). Hyperhomocysteinemia (HHcy) is an independent risk factor for CVDs, including AS. HHcy may participate in AS by regulating S-nitrosylation. Chen et al*.* reported that HHcy can promote AS by reducing endothelial or aortic protein S-nitrosylation levels (Chen et al. 2015b). In addition, Li et al*.* reported that HHcy could also reduce the level of protein S-nitrosylation in T cells, ultimately promoting the secretion of inflammatory cytokines and the proliferation of T cells and AS. Mechanistically, HHcy increased the expression of S-nitrosoglutathione reductase (GSNOR), a key enzyme controlling denitrosylation. These results provide new insights into HHcy-induced AS (Li et al. 2018b). Taken together, PTMs, including phosphorylation, glycosylation, S-nitrosylation, and SUMOylation, are instrumental in regulating AS development; however, detailed functions and mechanisms require further investigation to provide a basis for developing precise treatments.

### Role of PTMs in obesity

Obesity, a serious public health problem worldwide, is a significant risk factor for many diseases, including CVDs, T2DM, and non-alcoholic fatty liver disease (Wensveen et al. 2015). The pathogenesis of obesity is complicated and includes genetic factors, environmental factors, and metabolic dysregulation (Cruciani et al. 2023). Recent studies have explored the roles and molecular mechanisms of PTMs in obesity. For example, N-myristoylation is a ubiquitous, generally co-translational modification of newly synthesized proteins that involves attachment of the C14 fatty acid (myristic acid) to N-terminal glycine (Rampoldi et al. 2012). Neopane et al. reported that blocking AMPK β1 myristoylation enhanced AMPK activity and protected mice from high-fat diet-induced obesity and hepatic steatosis (Neopane et al. 2022). AMPK is a cellular energy sensor that can phosphorylate a variety of substrates, including key metabolic proteins and transcription factors, to restore energy homeostasis. Therefore, these results may provide a novel strategy for treating metabolic diseases. As mentioned above, FXR controls the expression of many genes involved in bile acid, lipid, glucose, and amino acid metabolism, and maybe a potential target for diseases associated with metabolic disorders. Numerous studies have shown that FXR can be modified by several PTMs and can affect obesity-related disorders. For example, Kim et al. revealed that a dysregulated acetyl/SUMO switch in FXR could promote obesity. Mechanistically, acetylation of FXR blocks its interaction with the SUMO ligase PIASy and inhibits SUMO2 modification at K277, leading to obesity (Kim et al. 2015). These results provide potential therapeutic and diagnostic targets for obesity-related metabolic disorders. Obesity is also a significant risk factor for kidney damage, namely obesity-related nephropathy (Arabi et al. 2022). Chen et al. showed that IκB kinase could inactivate the deubiquitination activity of cylindromatosis protein by activating its phosphorylation, thus promoting the ubiquitination of Nrf2 and aggravating oxidative stress injury in the kidney in obesity-related nephropathy (Chen et al. 2021b). In summary, PTMs play an important role in obesity and obesity-related diseases and represent a large number of potential therapeutic targets. However, in the future, more experimental studies are needed to explore the roles and mechanisms of various PTMs in regulating the expression of genes related to metabolism and the resulting impact on obesity.

### Role of PTMs in tumor

PTMs are crucial for controlling tumor immunity and immunotherapy and offering a potential target for enhancing the effectiveness of immunotherapy. Tumor immune microenvironments and the impact of the immune system, in addition to changes in cancer cells, are the primary factors in tumor initiation and development. PTMs such as phosphorylation, ubiquitination, acetylation, and glycosylation, are thought to be associated with tumorigenesis. For instance, epidermal growth factor receptor phosphorylation by PFKP (Y64) has been known to be involved in AKT activation and AKT-mediated phosphorylation of β-catenin (S552), promoting the glycolytic process in brain tumor growth (Lee et al. 2020). Glycyrrhizin inhibits HK2 by decreasing the phosphorylation level of AKT, suppressing the Warburg effect and cell proliferation in peripheral nerve injury (Sun et al. 2021a). PKC increases the phosphorylation and nuclear translocation of PKM2 to enhance lipogenesis and tumor development in prostate cancer cells (Lai et al. 2023). Moreover, tripartite motif-containing 35 (Trim35) regulates the tetrameric and dimeric leaps of PKM2 through ubiquitin action and affects the malignant biological behavior of breast cancer by regulating the Warburg effect (Wu et al. 2022). Glycosylation of PFK1 at (S529) reduces cancer cell proliferation in vitro and slows tumor development (Yi et al. 2012). PKM2 glycosylation may be a novel target for controlling cancer metabolism and tumorigenesis in colorectal cancer (Chaiyawat et al. 2015). Membrane-associated RING-CH 8 promotes ubiquitination-mediated proteasomal degradation to reduce HK2 protein levels, thereby regulating and repressing glycolysis to promote tumor suppressors in colorectal cancer (Wang et al. 2022e). Cisplatin-acetylated PFKFB3 (K472) causes accumulation of PFKFB3 in the cytoplasm, which facilitates its phosphorylation by AMPK, leading to PFKFB3 activation and enhanced glycolysis (Li et al. 2018a). Epidermal growth factor receptor activation rapidly increases PFKFB3 phosphorylation and expression and increases glycolysis in non-small cell lung cancer cells (Lypova et al. 2019). These studies reveal that cancer development, progression, and metastasis are intimately correlated with PTMs, although the underlying molecular pathways remain poorly understood. Further, PTM-mediated dysfunction of glucose and lipid metabolism, especially its effects on various organs, is closely related to tumorigenesis. Hence, PTMs can be highly relevant in the search for drug targets and diagnostic biomarkers in tumorigenesis.

### Role of PTMs in sepsis

Sepsis is defined as the presence of systemic signs of infection, while severe sepsis is defined as sepsis plus sepsis-induced organ dysfunction or tissue hypoperfusion (Singer et al. 2016). PTMs are significantly associated with sepsis-associated lung injury, myocardial injury, and encephalopathy. For instance, modulation of sepsis-enhanced glycolysis with 2-deoxy-d-glucose significantly attenuates sepsis-induced cardiac dysfunction. These mechanisms involve attenuating sepsis-induced pro-inflammatory responses and myocardial apoptosis by decreasing mitogen activated protein kinase 3 phosphorylation (Zheng et al. 2017). In addition, in the LPS-treated human umbilical vein endothelial cell (HUVEC) model, dichloroacetate restored pyruvate dehydrogenase complex function by reversing LPS-induced phosphorylation of pyruvate dehydrogenase E1 (S293 and S300), preventing lactic acid production and HUVEC monolayer barrier dysfunction (Mao et al. 2022). Moreover, inhibition of glycolysis or the prevention of PKM2 nuclear aggregation significantly reduces the phosphorylation and activation of transcription factor 2 (ATF2), thus reducing LPS-induced pyroptosis of microglia (Li et al. 2021). ER stress can increase the phosphorylation of signal transducer and activator of transcription 3 (STAT3) and monoclonal antibody to Suppressor of Mothers against Decapentaplegic (SMAD) family member 3 (Smad3), and also activate UPS‐mediated proteolysis to promote sepsis‐induced muscle atrophy (Zheng et al. 2023). Finally, p53 deacetylation by the deacetylase Sirtuin 1 (Sirt1) through resveratrol/quercetin administration or mutation of the acetylated lysine site in p53 promotes renal tubular epithelial cell autophagy, alleviating sepsis-induced acute kidney injury. Other PTM-mediated dysfunctions of glucose metabolism are closely related to sepsis. For instance, cynaroside inhibited glycolysis-related proteins, including PFKFB3, HK2, and HIF-1α, and glycolysis-related hyperacetylation of high mobility group box 1 (HMGB1) to restore PK activity in the septic liver (Pei et al. 2021). Furthermore, Hwang et al*.* reported a protective effect of glucosamine on sepsis, potentially through the O-GlcNAcylation of nucleocytoplasmic proteins in sepsis-induced lung injury and inflammation (Hwang et al. 2019). Therefore, PTMs exert various physiological effects in sepsis models by affecting the lung, skeleton, brain, and cardiac muscles. Overall, the most common PTMs involved in glucose metabolism include phosphorylation, acetylation, and ubiquitination.

## Further prospects of PTMs in glucose and lipid metabolism

PTMs play essential roles in cellular physiology and pathology, regulate glucose and lipid metabolism, and influence almost all aspects of cell biology and pathogenesis. However, many issues remain to be resolved before PTM sites can be used as promising targets for treating glucose and lipid metabolism disorders. Understanding the molecular mechanisms underlying PTMs could shed light on new therapeutic interventions. Although excellent work on PTMs has been carried out in past decades, PTMs of rate-limiting enzymes in glycosphingolipid biosynthesis need to be considered for future development. Moreover, PTMs have diverse functions and can regulate other PTMs, leading to complex regulatory crosstalk. Interprotein crosstalk between phosphorylation and SUMOylation has been widely reported. For example, S-phase kinase-associated protein 2, an E3 ubiquitin ligase, mediates FBP1 protein ubiquitination and degradation induced by phosphatase and tensin homolog loss and promotes the Warburg effect in prostate cancer cell growth (Song et al. 2022). Enhancing the connection between Akt and HK2 through K63-linked ubiquitination eventually leads to an increase in the phosphorylation of HK2 on Thr473 and mitochondrial localization, which is involved in glycolysis and tumor development (Yu et al. 2019). Thus, these PTMs greatly complicate mechanisms that modulate proteasome activity.

In addition, various regulations of rate-limiting enzymes in glucose and lipid metabolism could improve our understanding of the biological roles of these PTMs and provide a foundation for the research of regulatory mechanisms for these types of PTMs. Different diseases affect the corresponding processes of glycolipid metabolism, thus exerting specific regulatory effects. In diabetes, glucose transport disorders and gluconeogenesis have become the focus of disease intervention, and various glucose-lowering drug treatments have been developed based on the PTMs of SGLT and GLUT. However, in sepsis, PTMs specifically regulate metabolic changes in the septic state by modulating the activity and localization of enzymes, such as glycolytic processes, mainly affecting the Warburg effect, which has become an essential target for sepsis intervention. Angiogenesis and immune escape are important intervention targets for PTMs in tumor development. With the development of genomic, transcriptomic, proteomic, and epigenetic technologies, the prospects of novel drugs targeting PTM sites are promising. PTM sites have been proven to be promising therapeutic sites for treating glucose and lipid metabolism disorders, although further studies are needed to elucidate the mechanisms involved.

Advances in next-generation sequencing and mass spectrometry proteomics technologies have led to an explosion of data on PTM sites and disease-associated glucose and lipid metabolism. To predict succinylation sites, machine-learning-based prediction of protein modification sites, such as DeepSuccinylSite, has become popular (Thapa et al. 2020). In the future, PTM sites may become novel biomarkers and therapeutically-related targets for glucose and lipid metabolism diseases. For example, PTMs of blood-derived alpha-synuclein can act as biochemical markers for Parkinson’s disease (Vicente et al. 2017). Although research on PTMs has increased in recent years, their role in glucose and lipid metabolism disorders requires further investigation.

Thus far, proteomic studies on PTMs that regulate development have primarily focused on phosphorylation. Frequently, abnormal phosphorylation causes cellular processes to become disorganized, which ultimately results in the onset and progression of illnesses. Consequently, medications often target kinases and phosphatases. Nearly one-third of the pharmaceutical industry's current drug development initiatives focus on PKs, one of the most significant categories of therapeutic targets. For example, the hypoglycemic and anti-obesity characteristics of cardiotrophin-1 (CT-1) may be explained by the fact that CT-1 limits intestinal sugar absorption by lowering SGLT-1 levels through AMPK phosphorylation (Lopez-Yoldi et al. 2016). Additionally, by increasing the activities of HK, glycogen synthase, and the phosphorylation of glycogen synthase kinase 3 (GSK3) protein, whole-grain highland barley enhances glycogen storage in the liver (Deng et al. 2020). Similarly, other PTMs can be used as targets for drug therapy. The PI3-K/Akt-GSK3beta-FBW7 signaling axis was downregulated by xanthohumol, which led to the ubiquitination of c-Myc and inhibition of tumor glycolysis (Yuan et al. 2020). Further multicenter clinical studies are needed to emphasize the role of modifications in clinical applications and confirm their clinical significance.

## Conclusion

In this review, we summarized the latest advancements pertaining to PTMs involved in regulating glucose and lipid metabolism. Regulation of rate-limiting metabolic enzymes is essential for controlling cellular metabolic changes. PTMs offer a dynamic way to regulate subcellular localization, stability, and protein interactions and activity. Moreover, PTMs regulate cellular metabolism, especially involving rate-limiting metabolic enzymes. In recent years, PTMs have been shown to participate in nearly all aspects of vital biological processes by regulating protein functions, such as glucose transport, glycolysis, and gluconeogenesis, and aberrant states of PTMs are frequently implicated in diseases involving glucose and lipid metabolism. Hence, PTM sites may become potential therapeutic targets for regulating glucose and lipid metabolism and controlling disease progression. Therefore, in-depth insights into the mechanisms of PTMs in glucose and lipid metabolism may provide a theoretical basis for developing new drugs. Thus, future studies should focus on the following issues:

1. Molecular mechanisms underlying the role of AKT in PTMs of GLUT and SGLT transporter proteins.
2. Regulation of PFK by PTMs that affects the Warburg effect and glycolytic pathway in sepsis.
3. Thorough investigation of the expression of PC and regulation of its activity, as PC, in addition to PEPCK, FBP-1 and G6Pase, is another rate-limiting enzyme in gluconeogenesis.
4. Clinical research regarding precision medicine and potential therapeutic targets for clinical diagnosis, prognosis, and therapy of PTMs in lipid and glucose metabolism.
5. Improved understanding of the physiological effects of crosstalk between different PTMs.
6. Regulation of non-rate-limiting enzymes, in addition to the PTMs of critical enzymes involved in glycolipid metabolism.

## Data Availability

Not applicable.

## Abbreviations

ACC: Acetyl-CoA carboxylase
ATF2: Activation of transcription factor 2
AD: Alzheimer’s disease
AMPK: AMP-activated protein kinase
AS: Atherosclerosis
ATP: Adenosine triphosphate
CVDs: Cardiovascular diseases
CPT-I: Carnitine palmitoyl transferase
DUBs: Deubiquitinases
DM: Diabetes mellitus
ERAD: Endoplasmic reticulum-associated degradation
FXR: Farnesoid X receptor
FAO: Fatty acid oxidation
FAT: Fatty acid translocase
FATP: Fatty acid transport proteins
FABP: Fatty acid-binding protein
GLUT: Glucose transporters
HIF1α: Hypoxia-inducible factor 1 alpha
HK: Hexokinase
HUVEC: Human umbilical vein endothelial cell
KATs: Lysine acetyltransferases
KDACs: Lysine deacetylases
MYD88: Myeloid differentiation primary response 88
OGT: O-GlcNAc transferase
OGA: O-GlcNAcase
PEPCK: Phosphoenolpyruvate carboxykinase
PFK: Phosphofructokinase
PFK: Phosphofructokinase
PTMs: Post-translational modifications
PC: Pyruvate carboxylase
PK: Pyruvate kinase
AKT: Protein kinase B
SUMO: Small ubiquitin-like modifier
SGLTs: Sodium-dependent glucose transporters
TRIF: Toll/Interleukin-1 receptor-domain-containing adapter-inducing interferon-β
